# A Nationwide Saudi Oral Health-related Quality of Life Appraisal by OHIP-14: A Cross-sectional Study

**DOI:** 10.3290/j.ohpd.c_2666

**Published:** 2026-05-07

**Authors:** Diaa Almutairi

**Affiliations:** a Diaa Almutairi Consultant in Dental Public Health, Dental and Oral Health Department, Prince Sultan Military College of Health Sciences, Dhahran, Saudi Arabia. Investigation and data acquisition; interpreted the data; wrote the manuscript.

**Keywords:** dental public health, OHIP-14, OHRQoL, oral health, quality of life.

## Abstract

**Purpose:**

To assess the oral health–related quality of life (OHRQoL) across Saudi Arabia and identify the most affected domains and regions.

**Materials and Methods:**

A cross-sectional online survey was conducted using the Arabic-validated OHIP-14 questionnaire and sociodemographic data. OHIP-14 scores (0–56) indicate poorer OHRQoL at higher values. A total score ≥60% was defined as poor OHRQoL.

**Results:**

Among 694 participants, 70.6% were female and 66.4% were aged 25–44. The prevalence of poor OHRQoL was 14.8%. Physical pain (26.8%) was the most frequently reported domain, followed by psychological discomfort (25.2%) and psychological disability (23.5%). The mean OHIP-14 total score was 15.8 ± 14.4; physical pain showed the highest mean domain score (3.3 ± 2.2). Poor OHRQoL was statistically significantly more common in males, older adults (>44 years), those with lower education, and smokers (all p < 0.05). The Southern region had the highest median OHIP-14 score (27.0), followed by Northern (18.0) and Central (14.0), while the Eastern region had the lowest (6.0) (p < 0.001).

**Conclusion:**

Oral problems considerably affect the quality of life of Saudi adults, particularly in physical pain and psychological domains. Vulnerable groups include males, older individuals, smokers, and those with lower education, while the Southern and Northern regions show the highest impact. Targeted preventive and educational interventions are needed to address these disparities.

The field of dentistry has long acknowledged oral health as an integral component of general health and well-being. Oral health plays a crucial role in essential daily functions, such as eating, speaking, and social interaction.^13^ It has been demonstrated that poor oral health has a detrimental impact on people’s social, psychological, and physical functioning, which lowers their overall quality of life.^26,42^ Furthermore, the quantity and condition of remaining dentition may profoundly influence the quality of life.^38^ Exploring the relationship between oral health and quality of life can assist in identifying individuals at risk for of a diminished quality of life, and may also underscore the importance of optimal oral health for enhancing life satisfaction.^10,15^

In this context, the concept of oral health-related quality of life (OHRQoL) has emerged as a subset of quality of life that reflects a variety of factors, including comfort while eating, sleeping, and engaging in social interaction. It also reflects self-esteem and satisfaction with oral health. Globally, OHRQoL has gained increasing attention in both research and clinical practice, as it is concerned with the subjective impact of oral health conditions on daily living. Thus, it provides insights into the broader consequences of oral diseases beyond clinical indicators.^18,35,41^

Various instruments have been developed to measure OHRQoL. While some tools primarily assess the overall impact of oral conditions, others emphasize functional limitations and their social consequences.^18,20^ The oral health impact profile (OHIP) is a widely recognized tool with well-established psychometric properties, applied in both cross-sectional and longitudinal research. The original version includes 49 items covering seven domains: functional limitation, physical pain, psychological discomfort, physical disability, psychological disability, social disability, and handicap.^4,37^ A shorter form, the OHIP-14, was later developed and widely validated to measure these impacts across different populations.^2^

In Saudi Arabia, oral diseases such as dental caries, periodontal conditions, and tooth loss remain highly prevalent, affecting different age groups and geographic regions.^31^ Existing studies have primarily examined OHRQoL among older adults^1,5,6,9^ or patients attending various dental healthcare services.^7,10^ Nevertheless, evidence on the impact of oral disorders on OHRQoL in the general population remains scarce. Furthermore, variations in socioeconomic status, healthcare access, lifestyle factors, and cultural practices across provinces may be linked with disparities in oral health impacts on the quality of life.^12,14,16,17,18,19,20,21,22,23,24,25,26,27,28,29,30^ Therefore, it is believed that OHRQoL will vary considerably across different provinces in Saudi Arabia. Consequently, the identification of the provinces experiencing the greatest burden along with the most affected dimensions of OHRQoL will generate valuable evidence to guide policymakers and health authorities in developing equitable, patient-centered oral health strategies tailored to the needs of diverse populations across Saudi Arabia. Therefore, this study aimed to assess the oral health–related quality of life among the general population in Saudi Arabia, and to identify the most affected domains and geographic regions.

## MATERIALS AND METHODS 

### Study Design

This cross-sectional survey study was conducted all over Saudi Arabia for two months. Ethical approval was obtained from the Institutional Review Board of Prince Sultan Military College of Health Sciences, Dhahran (Approval number IRB-2025-DOH-046). Consent was obtained from all participants after an explanation of the study’s objectives and methods. The confidentiality of the participants’ data was maintained by keeping the questionnaire anonymous and ensuring proper storage of the data, which was accessed only by the investigator and used for the current study purposes only. The study was conducted according to the guidelines of the Declaration of Helsinki.

### Eligibility Criteria

The study recruited adult men and women from Saudi Arabia’s five main regions (Southern, Northern, Central, Eastern, and Western). Participants were included if they agreed to take part and gave informed consent before completing the survey, while those who refused participation or submitted incomplete questionnaires were excluded.

### Sample Size and Sampling Technique

The sample size was calculated using the formula: Z^2^ × p(1−p)/d^2^, where Z = 1.96 corresponding to a 95% confidence level, p represents the estimated population proportion, and d = 0.05 is the margin of error. Because previous studies evaluating OHRQoL in the Saudi population have shown wide variations in prevalence, this study adopted a conservative estimate of p = 0.50 (50%). Accordingly, the minimum required sample size was 377 participants. A snowball sampling technique was employed across the five main regions of Saudi Arabia to obtain a sample as representative as possible of the target population.

### Data Collection

An online survey was developed using Google Forms and distributed via social media platforms. The survey, administered in Arabic, comprised 26 items divided into two sections. The first section collected sociodemographic data, including age, gender, region of residence, marital status, household size, educational level, employment status, smoking habits, and working in the dental field. The second section consisted of the Oral Health Impact Profile (OHIP-14) questionnaire.

### Study Instrument 

The study employed the OHIP-14 questionnaire to evaluate OHRQoL. This instrument has been validated in Arabic, demonstrating strong reliability, with intraclass correlation coefficients between 0.75 and 0.90 and Cronbach’s alpha >0.74, indicating good internal consistency.^1,3,4^ It consists of 14 items distributed across seven domains: functional limitation, physical pain, psychological discomfort, physical disability, psychological disability, social disability, and handicap. Each domain is assessed by two questions rated on a five-point Likert scale (0 = never, 1 = hardly ever, 2 = occasionally, 3 = often, 4 = always), in which participants were asked how often they had encountered adverse effects in these areas over the past 12 months. An adverse impact on quality of life in any domain was recorded when one or both items were answered “often” or “always.” Scores for the 14 items are summed to produce an overall OHIP-14 score (0–56), with higher scores indicating poorer quality of life. The OHRQoL was classified into two levels: good, defined as a score below 60% of the total possible score (<34), and poor, defined as a score equal to or greater than 60% (≥34).

To assess the Arabic questionnaire’s adaptation and feasibility, the OHIP-14 was translated into Arabic using a standard forward–backward method. Two bilingual experts independently produced forward translations, which were reconciled into a single version. This was back-translated into English by two independent bilingual translators blinded to the original questionnaire. A committee of translators and linguistic experts reviewed all versions, resolved discrepancies, and produced a pre-final Arabic version. This was piloted on 10 participants to assess clarity and cultural relevance. Then, a pilot study was carried out among 50 Saudi populations. The reliability analysis demonstrated good internal consistency, with a Cronbach’s alpha of 0.881. Item-total correlations ranged between 0.151 and 0.757. Analysis of Cronbach’s alpha if items were deleted showed no improvement over the overall coefficient, indicating that all items contributed meaningfully to the scale. Construct validity was established using correlation coefficients. The total score of each domain was calculated, and then Kendall’s tau b correlation was applied between the questions included in each domain and the domain total score. All items of the questionnaire were checked one by one; all of them exhibited values ranging from 0.558 to 0.953. Furthermore, correlations between the total score and each subscale scale values between 0.457 to 0.957.

### Statistical Analysis 

Data were tabulated and analyzed using SPSS version 27 (IBM; Armonk, NY, USA). Qualitative variables were summarized as frequencies and percentages. The numerical data were checked for normal distribution through histograms, Q-Q plots, boxplots, skewness and kurtosis coefficients. Normally distributed data were represented as mean ± standard deviation, while skewed data were displayed as median and interquartile range. The relationships between sociodemographic characteristics of the participants and frequency of adverse impacts on quality of life were examined using the chi-squared test. The various dimensions of the OHRQoL were compared using one-way repeated measure ANOVA, followed by Bonferroni’s adjusted pairwise comparisons. Greenhouse-Geisser correction was adopted due to violation of the sphericity assumption. Comparison of the total OHIP-14 and the scores of the seven dimensions among different Saudi provinces was done by the Kruskal-Wallis test, followed by Bonferroni’s adjusted pairwise comparisons. p-values less than 0.05 were considered statistically significant.

## RESULTS

The study included 694 participants, most of whom were female (70.6%). Nearly two-thirds were aged 25-44 (66.4%), while only 5.5% were ≥55 years. More than half were married (55.5%), and the majority lived in households with four or more members (74.1%). Regarding education, 60.2% were graduates and 25.8% held postgraduate degrees. Most participants were employed (59.5%), whereas students (9.4%) and stay-at-home partners (9.1%) formed smaller groups. Non-smokers comprised 78.5% of the sample. Geographically, the largest proportion resided in the Eastern region (36.9%), followed by the Central (21.3%) and Northern (14.1%) regions. Only 11 (1.6%) of the respondents worked in the dental field (Table 1).

**Table 1:** Sociodemographic characteristics of the study participants (N = 694)

		N = 694	%
Gender	Female	490	70.6%
	Male	204	29.4%
Age, year	18–24	110	15.9%
	25–34	208	30.0%
	35–44	232	33.4%
	45–54	106	15.3%
	≥55	38	5.5%
Marital status	Single	309	44.5%
	Married	385	55.5%
Household (number of family members)	One	38	5.5%
	Two	61	8.8%
	Three	81	11.7%
	Four or more	514	74.1%
Level of education	Middle school or less	10	1.4%
	High school	87	12.5%
	Graduate	418	60.2%
	Postgraduate	179	25.8%
Employment status	Employed	413	59.5%
	Unemployed	54	7.8%
	Self-employed	49	7.1%
	Retired	50	7.2%
	Stayed home partner	63	9.1%
	Student	65	9.4%
Smoking status	Non-smoker	545	78.5%
	Smoker	149	21.5%
Saudi province	Eastern	256	36.9%
	Western	105	15.1%
	Northern	98	14.1%
	Southern	87	12.5%
	Central	148	21.3%
Working in the dental field	No	683	98.4%
	Yes	11	1.6%
N: number.			

Table 2 presents the prevalence of oral health–related adverse impacts on quality of life. Physical pain, such as a toothache or discomfort while eating, was the most common (26.8%), followed by psychological discomfort (25.2%) and psychological disability (23.5%). Frequently reported psychological issues included feeling self-conscious (22.6%), tense (21.2%), or embarrassed (20.9%), and difficulty relaxing (18.9%). Additionally, 19% of participants reported experiencing handicap, while social disability was observed in 18.9%. Overall, 103 (14.8%) experienced poor oral health-related quality of life.

**Table 2:** Prevalence of adverse impacts on quality of life due to problems with teeth or mouth in the preceding twelve months

	N = 694	%
Trouble pronouncing	5	0.7%
Worsened taste	27	3.9%
**Functional limitation**	32	4.6%
Painful aching in the teeth	148	21.3%
Discomfort when eating	139	20.0%
**Physical Pain**	186	26.8%
Being self-conscious	157	22.6%
Feeling tense	147	21.2%
**Psychological discomfort**	175	25.2%
Unsatisfactory diet	108	15.6%
Interrupted meals	118	17.0%
**Physical disability**	125	18.0%
Difficulty relaxing	131	18.9%
Feeling embarrassed	145	20.9%
**Psychological disability**	163	23.5%
Irritated with other people	123	17.7%
Difficulty doing usual jobs	123	17.7%
**Social disability**	131	18.9%
Felt life is less satisfying	130	18.7%
Totally unable to function	115	16.6%
**Handicap**	132	19.0%
**Total OHIP-14 Score**		
**Good**	591	85.2%
**Poor**	103	14.8%
NB: The reported numbers for items and domains represent “often” or “always” responses.		

Table 3 demonstrates that males, older individuals (>44 years), those with lower education, and smokers experienced statistically significantly higher frequencies of adverse impacts on oral health–related quality of life (all p-values <0.05). Being male was statistically significantly associated with functional limitation (10.3%), physical pain (33.3%), physical disability (26.5%), social disability (24.5%), and handicap (25.0%). Older individuals (>44 years) showed statistically significantly higher prevalence of functional limitation (9.0%), psychological disability (31.9%). The lower education group was statistically significantly associated with functional limitation (5.5%), physical pain and disability (37.1% and 28.9%), psychological discomfort and disability (42.3% and 31.9%), and social disability and handicap (29.9% and 30.9%). Finally, smoking showed statistically significant associations with functional limitation (9.4%) and social disability (25.5%).

**Table 3:** Associations between frequency of adverse impacts on quality of life and sociodemographic characteristics (N= 694)

	Gender		Age in years		Education level		Smoking	
	Female	Male	≤44	>44	Level 1	Level 2	Non-smoker	Smoker
**Functional limitation**	11	21	19	13	15	17	18	14
	2.2%	10.3%*	3.5%	9.0%*	15.5%*	2.8%	3.3%	9.4%*
**Physical pain**	118	68	139	47	36	150	138	48
	24.1%	33.3%*	25.3%	32.6%	37.1%*	25.1%	25.3%	32.2%
**Psychological discomfort**	116	59	130	45	41	134	133	42
	23.7%	28.9%	23.6%	31.3%	42.3%*	22.4%	24.4%	28.2%
**Physical disability**	71	54	95	30	28	97	90	35
	14.5%	26.5%*	17.3%	20.8%	28.9%*	16.2%	16.5%	23.5%
**Psychological disability**	109	54	117	46	33	130	124	39
	22.2%	26.5%	21.3%	31.9%*	34.0%*	21.8%	22.8%	26.2%
**Social disability**	81	50	101	30	29	102	93	38
	16.5%	24.5%*	18.4%	20.8%	29.9%*	17.1%	17.1%	25.5%*
**Handicap**	81	51	99	33	30	102	99	33
	16.5%	25.0%*	18.0%	22.9%	30.9%*	17.1%	18.2%	22.1%
*Statistically significant at p<0.05. Level 1: no college degree; Level 2: college degree or postgraduate education.								

Table 4 shows a mean total OHIP-14 score of 15.8 ± 14.4, and a mean physical pain score of 3.3 ± 2.2, which was the highest, followed by psychological discomfort (2.7 ± 2.6) and psychological disability (2.5 ± 2.5). Alternatively, functional limitation showed the lowest mean score (1.1 ± 1.4). The repeated measures ANOVA showed a statistically significant difference across the dimensions (p < 0.001). Pairwise comparisons revealed that all dimensions were statistically significantly different from each other (p < 0.001), except for physical disability versus social disability and handicap (p = 1.00), and social disability versus physical disability (p = 1.00), indicating some overlap between these specific impacts on quality of life.

**Table 4:** Comparison between various dimensions of the Oral Health-Related Quality of Life among the study participants (N=694)

Quality of life dimensions	Mean ±SD	Greenhouse-Geisser repeated measures ANOVA	Bonferroni-adjusted pairwise comparisons between the dimensions
Total OHIP-14 score	15.8± 14.4		
Functional limitation	1.1±1.4	p<0.001*	All p-values <0.001*
Physical pain	3.3±2.2		All p-values <0.001*
Psychological discomfort	2.7±2.6		All p-values <0.001*
Physical disability	2.1±2.5		All p-values <0.001* except in dimensions 6 and 7 (p=1.00)
Psychological disability	2.5±2.5		All p-values <0.001*
Social disability	2.1±2.6		All p-values <0.001* except in dimension 4 (p=1.00)
Handicap	2.1±2.6		All p-values <0.001* except in dimension 6 (p=1.00)
*Statistically significant at p<0.05. ANOVA: one-way analysis of variance; SD: standard deviation; OHIP: Oral Health Impact Profile.			

Table 5 illustrates comparisons of the median scores of the total OHIP-14 and its seven dimensions across five Saudi provinces: Eastern, Western, Northern, Southern, and Central. The Southern province showed the highest median total OHIP-14 score (27.0), indicating the greatest oral health impact, followed by the Northern (18.0) and Central (14.0) provinces, while the Eastern province had the lowest median score (6.0) (p < 0.001). Statistically significant differences were also found across all individual dimensions, with Southern province consistently showing higher median scores, such as 4.0 for physical pain, psychological discomfort, psychological disability, social disability, and handicap, compared to lower scores in Eastern and Western provinces.

**Table 5:** Comparison of the total OHIP-14 and the seven dimensions’ scores among different Saudi Provinces

		Saudi Province					p-value
		Eastern	Western	Northern	Southern	Central	
Total OHIP-14 score	Median	6.0	12.0	18.0	27.0	14.0	<0.001*
	IQR	3.0-13.0	4.0-33.0	9.0-26.0	13.0-37.0	5.0-29.5	
p1 < 0.001*, p2 < 0.001*, p3 < 0.001*, p4 < 0.001*, p5 = 0.861, p6 = 0.003*, p7 = 1.00, p8 = 0.549, p9 = 1.0, p10 = 0.006*							
Functional limitation	Median	0.0	1.0	1.0	1.0	0.0	<0.001*
	IQR	0.0-2.0	0.0-2.0	0.0-3.0	0.0-2.0	0.0-2.0	
p1 = 0.001*, p2 < 0.001*, p3 = 1.0, p4 = 1.0, p5 = 1.0, p6 = 0.675, p7 = 0.007* p8 = 0.215, p9 = 0.001*, p10 = 1.0							
Physical pain	Median	2.0	3.0	4.0	4.0	4.0	<0.001*
	IQR	1.0-4.0	1.0-5.0	2.0-5.0	2.0-6.0	2.0-5.0	
p1 = 0.004*, p2 < 0.001*, p3=1.0, p4 =1.0, p5 = 1.0, p6= 0.155, p7 = 1.0, p8=1.0, p9 = 1.0, p10=1.0							
Psychological discomfort	Median	1.0	2.0	4.0	4.0	2.0	<0.001*
	IQR	0.0-3.0	0.0-6.0	10.0-4.0	20.0-6.0	0.0-6.0	
p1 = 0.001*, p2 < 0.001*, p3 < 0.001*, p4 < 0.001*, p5 = 1.0, p6 = 0.004*, p7 = 1.0, p8 = 0.294, p9 = 1.0, p10 = 0.009*							
Physical disability	Median	0.0	1.0	2.5	4.0	1.0	<0.001*
	IQR	0.0-1.0	0.0-5.0	10.0-4.0	20.0-6.0	0.0-4.0	
p1 < 0.001*, p2 < 0.001*, p3 < 0.001*, p4 < 0.001*, p5 = 1.0, p6 = 0.009*, p7 = 1.0, p8 = 0.332, p9 = 1.0, p10 = 0.001*							
Psychological disability	Median	1.0	2.0	3.0	4.0	2.0	<0.001*
	IQR	0.0-2.0	0.0-6.0	10.0-4.0	20.0-6.0	0.0-6.0	
p1 < 0.001*, p2 < 0.001*, p3 < 0.001*, p4 < 0.001*, p5 = 1.0, p6 = 0.001*, p7 = 1.0, p8 = 0.031*, p9 = 1.0, p10 = 0.005*							
Social disability	Median	0.0	2.0	2.0	4.0	2.0	<0.001*
	IQR	0.0-1.0	0.0-6.0	0.0-4.0	20.0-6.0	0.0-6.0	
p1 < 0.001*, p2 < 0.001*, p3 < 0.001*, p4 < 0.001*, p5 = 1.0, p6 = 0.001*, p7 = 1.0, p8 = 0.001*, p9 = 1.0, p10=<0.001*							
Handicap	Median	0.0	2.0	2.0	4.0	2.0	<0.001*
	IQR	0.0-1.0	0.0-5.0	0.0-4.0	20.0-6.0	0.0-4.5	
p1 < 0.001*, p2 < 0.001*, p3 < 0.001*, p4 < 0.001*, p5 = 1.0, p6 = 0.001*, p7 = 1.0, p8 = 0.019*, p9 = 1.0, p10=<0.001.*							
*Statistically significant at p<0.05, IQR: interquartile range, P1: Eastern vs Western, p2: Eastern vs Northern, p3: Eastern vs Southern, p4: Eastern vs Central, p5: Western vs Northern, p6: Western vs Southern, p7: Western vs Central, p8: Northern vs Southern, p9: Northern vs Central, p10: Southern vs Central. Comparisons were performed using the Kruskal-Wallis test, followed by Bonferroni-adjusted pairwise comparisons.							

## DISCUSSION

Oral health-related quality of life (OHRQoL) is a key component of overall health and well-being, reflecting how oral conditions affect physical, psychological, and social functioning.^8^ Compromised oral health not only diminishes personal quality of life but also contributes to lost productivity and a greater burden on healthcare systems through increased demand for preventive, restorative, and emergency dental services. Promoting and maintaining good oral health is crucial for safeguarding long-term health outcomes and minimizing the burden on public health resources.^27,32^

This study provides insight into oral health–related quality of life (OHRQoL) of the Saudi population, revealing that 103 of the 694 participants (14.8%) had poor OHRQoL. Comparable research among Saudi university students reported a slightly higher prevalence (17.1%),^39^ while another Saudi study focusing on older adults found a substantially greater burden (47.5%).^1^ In Riyadh, the use of the Arabic version of the Geriatric Self-Efficacy Scale for Oral Health showed that 11.14% of elderly participants had poor overall quality of life.^9^ Internationally, a study from New Zealand by Hong et al^23^ investigated OHRQoL among middle-aged individuals, reporting poor quality of life in 23.2%, 22.6%, and 24.2% of participants aged 32, 38, and 45, respectively.

The mean OHIP-14 score in this study was 15.8 ± 14.4, almost matching findings from earlier Saudi studies conducted among both elderly individuals^1^ and young adults.^39^ A systematic review and meta-analysis on OHRQoL in older populations reported a comparable mean score of 16.2.^11^ In contrast, a substantially lower average (6.41 ± 7.05) was observed among Brazilian older adults.^14^ These variations may result from differences in access to dental services, oral health awareness, or the demographic composition of the samples studied.

Physical pain was identified as the most frequently affected domain, with 26.8% of participants reporting frequent pain related to dental problems. This was followed by psychological discomfort (25.2%) and psychological disability (23.5%). Negative effects on social life (18.9%) and overall life satisfaction (19%) were also noted, whereas functional limitation was the least affected area, with only 4.6% experiencing problems with speech or taste. These results are consistent with findings from other studies: a recent investigation among older Saudi adults reported physical pain, physical disability, and psychological disability as the most impacted domains^1^. A survey of Saudi university students^39^ and Jordanian patients having periodontal disease showed a similar pattern.^3^ Likewise, research conducted in Jazan, Saudi Arabia, found that physical pain and psychological discomfort had the highest OHIP scores.^22^ The Indonesian population showed the greatest impact in the physical pain domain,^24^ while Sudanese adults ranked psychological discomfort first among all domains of OHRQoL.^28^ An alternative pattern was explored by Papaioannou et al,^31^ who studied Greek adults from rural and urban areas and discovered elevated scores in functional limitation, physical pain, and handicap domains.

Regarding sociodemographic differences in OHRQoL, males, older adults, those with lower educational attainment, and smokers reported statistically significantly higher frequencies of negative impacts. The poorer OHRQoL observed among men is consistent with findings from a Saudi study of young adults, which showed that males had 1.89 times higher odds of poor OHRQoL than did females.^39^ However, this contrasts with research from Russia,^21^ as well as Sudan,^28^ where women demonstrated statistically significantly lower OHRQoL. A study in China by Lu et al^29^ found a similar distribution between the sexes. Such discrepancies may be linked to reduced use of dental services or higher tobacco consumption among men.

The finding that older adults showed greater negative impacts, mainly functional limitation and psychological disability, aligns with previous studies^1,3,7,8,10,11,12,13,14,15,16,17,18,19,20,21,23^ and is consistent with evidence that age increases the risk of caries, tooth loss, and periodontal problems.^17^ Oral functions, such as saliva production, may also be impaired, resulting in xerostomia and tooth loss, which in turn can negatively influence chewing and swallowing.^25^ Moreover, inadequate oral hygiene heightens the likelihood of developing dental problems, including caries and periodontal disease.^19^

Another important finding of this study was the influence of educational level on OHRQoL. Participants without a university degree showed statistically significantly poorer outcomes across all dimensions, including functional limitation, physical pain and disability, psychological discomfort and disability, as well as social disability and handicap. Regular dental screening has been shown to play an important role in enhancing oral health^36^. In Saudi Arabia, the Ministry of Health has implemented a dental screening program for schoolchildren; however, no structured screening initiatives currently exist within university settings for students.

Smokers experienced greater negative impacts, consistent with the well-established link between tobacco use and oral conditions such as periodontal disease, tooth loss, and general discomfort. Numerous studies have similarly demonstrated a statistically significant association between smoking and poorer OHRQoL.^1,12,34^ Thirunavukkarasu et al^39^ further found that smokers had 3.47 times higher odds of experiencing poor OHRQoL. This relationship may be explained by the harmful effects of smoking on oral tissues, including reduced blood flow, increased local edema, and inflammation.^40^

A salient aim of the present study was the investigation of the regional differences in the impact of oral conditions on quality of life. Participants from the Southern and Northern provinces experienced the greatest impact, whereas those from the Eastern region reported the least. These differences highlight potential regional disparities in oral health. They also could reflect unequal access to dental services, variations in the availability of preventive programs, or differences in socioeconomic status, dietary patterns, and health literacy between regions. Cultural attitudes toward oral health may also contribute, with populations in some areas perhaps being less likely to seek routine dental care, leading to more advanced disease and greater discomfort. Additionally, geographic challenges such as limited transportation or fewer specialized clinics in remote areas might explain the higher burden in the Southern and Northern provinces. Addressing these disparities may require improved workforce distribution, mobile dental services, and community-based education initiatives to promote preventive practices and timely treatment.

### Limitations

Although the study included a relatively large and geographically diverse sample of the Saudi population and utilized the validated OHIP-14 instrument for data collection, some limitations should be noted. Its cross-sectional design precludes causal inference, and reliance on self-reported information introduces the risk of recall bias. In addition, the use of online, non-probability sampling may have led to selection bias, potentially limiting the generalizability of the results to the broader population.

## CONCLUSION

The study findings indicate that that oral conditions negatively affect the quality of life of the Saudi population, with physical pain and psychological discomfort emerging as the most affected domains with the highest OHIP scores. Poor OHRQoL was associated with male gender, older age, lower educational attainment, and smoking, while participants from the Southern and Northern provinces exhibited the greatest burden. These findings highlight the importance of preventive measures and educational initiatives aimed at managing pain and supporting psychological well-being. Targeted interventions for smokers, men, and individuals with limited education, along with efforts to reduce regional disparities in service accessibility, could enhance OHRQoL at the population level.

## REFERENCES 
